# Visualization of translation reorganization upon persistent ribosome collision stress in mammalian cells

**DOI:** 10.1016/j.molcel.2024.01.015

**Published:** 2024-03-21

**Authors:** Juliette Fedry, Joana Silva, Mihajlo Vanevic, Stanley Fronik, Yves Mechulam, Emmanuelle Schmitt, Amédée des Georges, William James Faller, Friedrich Förster

**Affiliations:** 1 Structural Biochemistry, Bijvoet Centre for Biomolecular Research, Utrecht University, 3584 CG Utrecht, the Netherlands; 2 MRC Laboratory of Molecular Biology, Cambridge, CB2 0QH, UK; 3 Division of Oncogenomics, The Netherlands Cancer Institute, Amsterdam, the Netherlands; 4 Laboratoire de Biologie Structurale de la Cellule, BIOC, Ecole polytechnique, CNRS, Institut Polytechnique de Paris, 91128 Palaiseau Cedex, France; 5 Structural Biology Initiative, CUNY Advanced Science Research Center, City University of New York, New York, NY, USA; 6 Department of Chemistry and Biochemistry, The City College of New York, New York, NY, USA; 7 Ph.D. Programs in Chemistry and Biochemistry, The Graduate Center, City University of New York, New York, NY, USA

**Keywords:** ribosome collision, cryoelectron tomography, translation regulation, polysome, initiation, ribosome quality control

## Abstract

Aberrantly slow ribosomes incur collisions, a sentinel of stress that triggers quality control, signaling, and translation attenuation. Although each collision response has been studied in isolation, the net consequences of their collective actions in reshaping translation in cells is poorly understood. Here, we apply cryoelectron tomography to visualize the translation machinery in mammalian cells during persistent collision stress. We find that polysomes are compressed, with up to 30% of ribosomes in helical polysomes or collided disomes, some of which are bound to the stress effector GCN1. The native collision interface extends beyond the *in vitro-*characterized 40S and includes the L1 stalk and eEF2, possibly contributing to translocation inhibition. The accumulation of unresolved tRNA-bound 80S and 60S and aberrant 40S configurations identifies potentially limiting steps in collision responses. Our work provides a global view of the translation machinery in response to persistent collisions and a framework for quantitative analysis of translation dynamics *in situ*.

## Highlights

• The native disome interface extends to the L1 stalk and eEF2
• Collisions vary in composition and conformation over time, with some bound to GCN1
• Saturation of the RQC leads to accumulation of non-functional tRNA-bound 60S complexes
• The accumulation of aberrant 40S complexes indicates an impairment of initiation

## Graphical abstract

**Figure:**
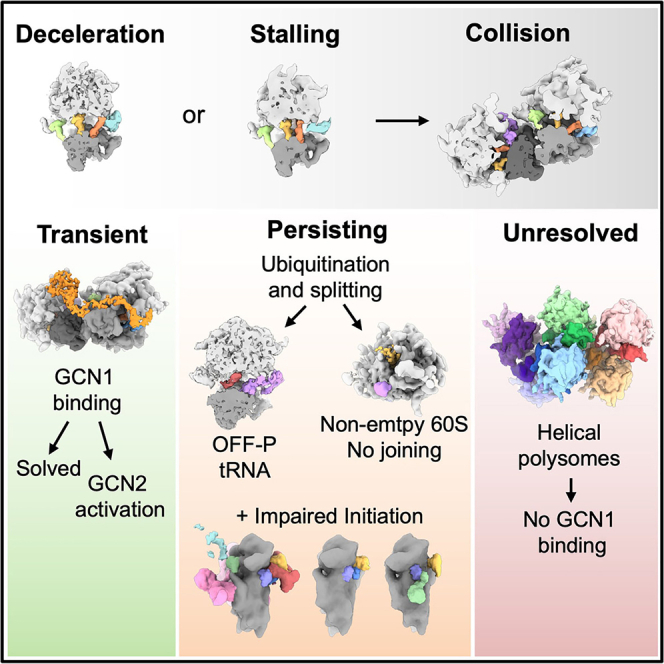

## Teaser

In cells, millions of ribosomes translate mRNAs into functional proteins. Ribosomes stalling during translation generate collisions that trigger a wide range of responses to avoid disease. Fedry et al. visualized how key constituents of these pathways change with collision stress over time.

## Introduction

**Figure 1:**
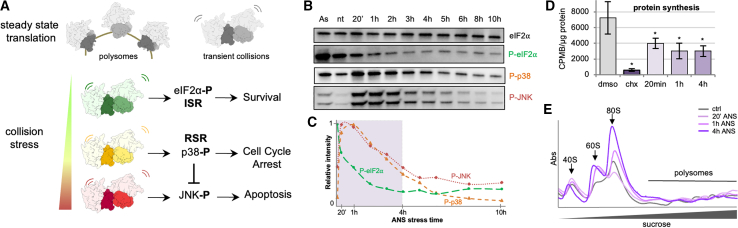
Biochemical analysis of low-dose ANS persistent collision stress in MEF cells (A) Schematic representation of translational situation in untreated cells (polysomes and low amount of collisions) and under increasing intensities of collision stress, with associated cellular stress responses and cell fate outcome. (B) Western blot analysis of collision-stress-induced responses in MEF cells treated with 500 μM arsenite for 10 min, untreated, or treated with 200 nM ANS for 20 min to 10 h: total eIF2α, phosphorylated eIF2α, phosphorylated p38, and phosphorylated JNK. (C) Corresponding relative intensity measurements, background substracted and normalized by total eIF2α intensity. (D) ^35^S incorporation protein synthesis measurements in control cells and cells treated with high-dose cycloheximide (CHX, 100 mg/mL), and low-dose ANS (200 nM) for 20 min (p = 0.049), 1 h (p = 0.03), and 4 h (p = 0.03). Error bars are standard deviations on n = 3 independent replicates. (E) Polysome profiling on sucrose gradients for control cells and cells treated with 20 min, 1 h, and 4 h low-dose ANS. See also Figure S1.

Translation of mRNA into protein is the last and decisive step of gene expression. It is one of the most energetically costly cellular processes for most cells. Hence, translation is not only tightly regulated but also carefully monitored for problems. A large class of problems occur during translation elongation, causing the ribosome to slow excessively or to stall. Potential causes of ribosome slowing include damaged or inappropriately processed mRNA, amino acid insufficiency, rare codons, ribosome-nascent chain interactions, damaged ribosomes, elongation factor dysfunction, toxins, and others. An immediate consequence of an aberrantly slow ribosome is collision of the trailing ribosome behind it (Figure 1A). Although occasional transient collisions might be normal on highly translated mRNAs, persistent collisions are an indicator of elongation stalling. Thus, cells use ribosome collisions as a key sentinel of aberrant translation and have evolved several mechanisms to resolve and respond to this situation.

Key responses to ribosome collisions include inhibition of further translation initiation on that mRNA, disassembly of collision complexes, degradation of the associated mRNA, degradation of the nascent proteins, recycling of ribosomal subunits, and signaling of multiple stress responses. Each of these responses has been characterized largely in isolation, resulting in considerable insights into the key required factors and their mechanisms of action. Structural analyses of purified collided ribosomes *in vitro* show a leading stalled ribosome in an unrotated state containing a canonical P-site tRNA abutting a collided ribosome trapped in the rotated-2 state.^1^^,^^2^^,^^3^^,^^4^^,^^5^^,^^6^ This distinctive collided di-ribosome (hereafter disome) architecture is thought to be recognized by various collision-specific factors to initiate downstream reactions.

EDF1 binds at the mRNA entry channel on the 40S subunit near the collision interface and recruits the translation repression factors GIGYF2 and eIF4E2 to prevent further initiation on the problematic mRNA.^7^^,^^8^ GCN1 binds both the leading and trailing ribosome of the collided disome and activates GCN2, a key effector of the integrated stress response (ISR) that attenuates global translation.^6^ The long isoform of ZAKα also recognizes stalled ribosomes and initiates the ribotoxic stress response (RSR),^9^^,^^10^ leading to cell cycle arrest and apoptosis.^10^^,^^11^ The E3 ligase ZNF598 selectively binds collided disomes and ubiquitinates RPS10,^1^^,^^3^^,^^12^^,^^13^ a signal for activation of the ASC-1 complex (ASCC) helicase.^14^^,^^15^^,^^16^ ASCC is thought to pull on the mRNA and split the stalled ribosome into 60S and 40S subunits.^17^ The peptidyl-tRNA-containing 60S complex is acted on by the ribosome quality control (RQC) pathway that leads to proteasomal degradation of the truncated nascent chain,^18^^,^^19^ whereas the 40S-mRNA complex engages mRNA degradation factors.

These and yet other collision-specific pathways are unlikely to be triggered in all circumstances or on all collisions. Instead, it is speculated that the response is hierarchical, with relatively conservative responses preceding more global responses, such as stress pathway activation (Figure 1A). At present, the amplitude and kinetic barriers of these various responses, their spatial relationships, and their combined effects on the translation and quality control machinery in cells is unknown. To begin addressing this problem, we have visualized and classified ribosome populations in mammalian cells experiencing collision stress using *in situ* cryoelectron tomography (cryo-ET). This approach preserves biochemically labile complexes, provides an unbiased view of the native state of translation, and affords the potential for extracting spatial information of parallel biochemical pathways. Among the many observations, our results reveal that *in vivo*: (1) binding of a tRNA in the Z site is favored on slow or stalled ribosomes; (2) collided disomes have a larger interface than previously known; (3) a highly efficient ZNF598/ASCC disassembly system limits collision accumulation and generates 80S monosomes containing a P-site tRNA; (4) clearance of 60S complexes is limited by Listerin availability; and (5) accumulation of 40S complexes in aberrant states indicative of impaired initiation. This study provides a foundation and roadmap for the *in situ* analysis of translation under a range of physiologic and pathologic states.

## Results and discussion

### Low-dose ANS induces stress responses and affects translation in MEF cells

To induce collision stress, we exposed mouse embryo fibroblast (MEF) cells to sub-inhibitory concentrations of the elongation inhibitor anisomycin (200 nM ANS) for between 20 min and 10 h, when cell death starts to occur (Figures 1B, 1C, and S1A–S1D). Western blot analysis revealed an early RSR response, with increased phosphorylation of the RSR markers p38 and JNK at 20 min of collision stress, gradually returning to baseline levels (Figures 1B and 1C). In contrast, the ISR marker phospho-eIF2α did not significantly increase during acute collision stress, unlike a positive control (500 μM arsenite for 10 min). Instead, eIF2α was progressively dephosphorylated over time (Figures 1B and S1E), a result also seen in U2OS cells (Figure S1F). Reduced eIF2α phosphorylation may be a compensatory response to decreased protein synthesis in the presence of ANS, which was about 2-fold lower than untreated cells, as measured by ^35^S-methionine incorporation (Figure 1D).

Polysome profiling revealed increased 80S and 60S complexes in stressed cells over time (Figure 1E). As we show later, the 80S monosomes seem to be derived from dissociation of ribosome collisions, with the 60S subunits presumably resulting from subsequent 80S splitting. Western blot analysis of polysome fractions demonstrated an accumulation of EDF1 over time (Figure S1H), consistent with the polysomes containing collided ribosomes. Although we did not detect increased ZNF598 on polysomes under our fractionation conditions (Figures S1G–S1I), its collision-specific activity was evident by increased RPS10 ubiquitination, as shown before.^7^^,^^8^ These results illustrate that MEF cells respond to low-dose ANS with a time-dependent and multi-tiered response that includes the RSR, reorganization of translation machinery and factors, and collision detection by EDF1 and ZNF598. This provides an experimental system for analyzing changes to the translation machinery *in situ* by applying cryo-ET to MEF cells before and after induction of ANS-triggered collision stress.

### The Z-site tRNA is a feature of stalled ribosomes

**Figure 2:**
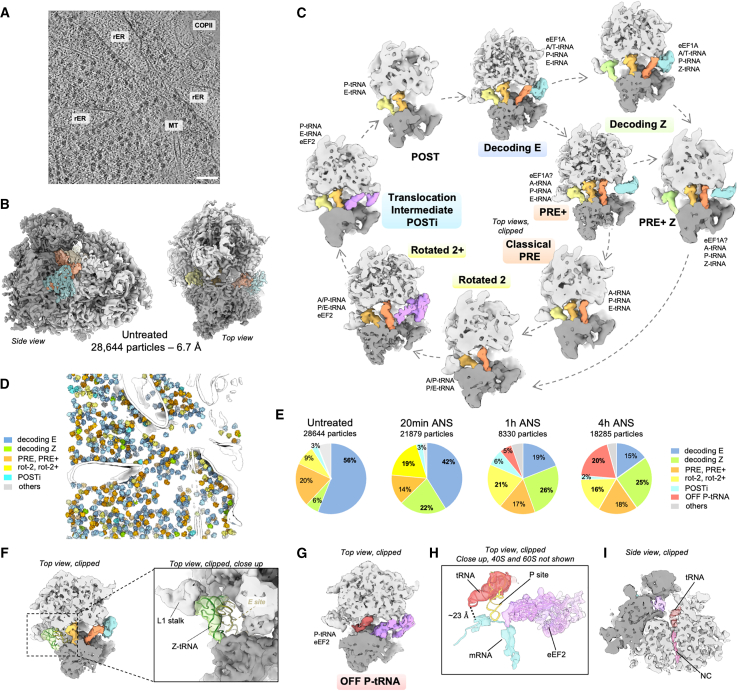
*In situ* visualization of 80S ribosome populations (A) Slice through a representative denoised tomogram of control MEF cells, scale bars: 100 nm. (B) Subtomogram average of 80S particles in the control condition. The small subunit is displayed in dark gray, the large subunit in light gray, the elongation factor in cyan and the tRNAs in shades of orange to yellow. (C) Observed active intermediates positioned in model of mammalian elongation cycle. The ribosome is clipped for visualization. A, P, and E indicate ribosomal aminoacyl, peptidyl, and exit sites, respectively. The tRNAs are color coded with respect to a complete cycle. The color code is the same as in (B), with eEF1A in cyan, eEF2 in purple, and the Z tRNA in green. (D) Different ribosome elongation states mapped back in the original tomogram shown in (A). Segmented membranes and microtubules are displayed in white. (E) Relative abundance of ribosomal elongation complexes in all datasets. (F) Close-up view on the Z-site-bound tRNA of the decoding Z complex. (G) Off-pathway ribosomal complex observed under prolonged low-dose ANS stress (1 and 4 h). The tRNA is displayed in dark red. (H) Same complex as in (G), displaying a model fit for the tRNA, eEF2, and the mRNA density (dark cyan). (I) Same complex as in (G) and (H), side view, clipped for the visualization of the peptide exit tunnel displaying a nascent chain (pink) bound to the tRNA. See also Figure S3.

Cryo-ET of untreated MEF cells resulted in 28,644 ribosomal particles and an 80S subtomogram average at ∼6.7 Å resolution (Figures 2A and 2B). To increase particle numbers and obtain a resolution benchmark for our setup, we combined these control particles with the 4 h ANS dataset (18,285 particles), achieving a resolution of ∼4.5 Å in the best-resolved large subunit (LSU) region (Figure S2). Using image classification (Figure S2), we identified nine 80S ribosome states in untreated cells, largely associated with polysomes. Seven of these states correspond to well-established eukaryotic 80S elongating complexes^20^^,^^21^^,^^22^ (Figures 2C–2E and S2): a decoding E-state (16,035 particles, 8.8 Å resolution)^20^^,^^23^^,^^24^; a classical PRE state, with or without elongation factor density (130–2,183 particles per class, resolution 26–9.2 Å)^20^^,^^21^^,^^23^^,^^25^^,^^26^; a rotated-2 state, without or with eEF2 (409 particles, 15.1 Å, rotated 2, and 2,184 particles, 9.8 Å, rotated-2+)^20^^,^^21^^,^^22^^,^^26^; an unrotated translocation intermediate (663 particles, 13.9 Å, POSTi)^20^^,^^21^; and a low-abundance unrotated POST state (178 particles, 19.7 Å).^20^^,^^21^ Two additional classes of 80S complexes on polysomes resemble decoding and PRE states but with tRNA absent from the E site. Instead, a tRNA was bound further out, in contact with the ribosome head and L1 stalk, previously referred to as the Z site^27^ (1,722 particles, 10.3 Å, decoding Z, Figures 2B and 2F) and (382 particles, 15.4 Å, PRE+ Z, Figures S3A–S3F). The significance of these Z-tRNA-containing classes became apparent when this baseline dataset was compared with cells experiencing collision stress.

To analyze the consequences of collision stress, we focused primarily on the 4 h ANS time point, when cells have substantially changed their translation state, initiated the RSR and RQC, but are not undergoing cell death (Figures 1B, 1C, and 1E). We compared this state of persistent collision stress to untreated cells, earlier time points of collision stress, and cells treated with high-dose ANS (for 3.5 h), which stalls all ribosomes before they can collide.^1^ We found that all major ribosomal populations observed in untreated cells were also present in stressed cells but that their relative ratios changed over time (Figures 2E and S3G–S3I). The rotated populations increased markedly, from 9% in untreated cells to 16%–21% in collision-stressed cells, and were nearly absent in cells where ribosomes were fully stalled with high-dose ANS. This observation is consistent with *in vitro* studies showing that collided ribosomes display the rotated-2 conformation.^1^^,^^2^^,^^3^^,^^4^^,^^5^^,^^6^

The decoding Z state seen in 6% of 80S ribosomes in untreated cells increased to ∼25% with collision stress, where classical PRE+ states are also found with a Z-site-bound tRNA. Neighbor analysis showed that in both untreated and collision stress conditions, the PRE+ Z classes have stronger density for a downstream ribosome compared with an upstream one (Figures S3A–S3F). This suggests that the PRE+ Z state corresponds to a stalled ribosome, which would be more likely to incur a collision and hence have a close downstream neighbor. The PRE+ class, displaying A- and P-site tRNAs, is strongly enriched under stalling stress (72% of total 80S, Figures S3H and S3I), which rationalizes previous ribosome profiling results where ANS stalling caused the accumulation of a 28-nt footprint associated with an occupied 40S A-site.^28^ In addition, these stalled PRE+ ribosomes also display a Z site, indicating that a collision is not a prerequisite for achieving a Z-site state. Although this state may have been missed in earlier reconstructions of isolated ribosomes due to the biochemical lability of the Z-site tRNA, it was also observed *in situ* in untreated HEK cells, but not on ribosomes stalled at the initiating AUG with homoharringtonin.^29^ This argues against Z-tRNA resulting from cytosolic deacylated tRNA rebinding to ribosomes, as previously proposed.^27^ Instead, the collective data so far suggests that Z-tRNA is favored on transiently or fully stalled ribosomes, and the observed subset of ribosomes in the PRE+ Z state in untreated MEFs, together with an enriched downstream neighboring ribosome, underscore the notion that ribosome stalling and collision occur at an appreciable frequency, even under normal growth conditions.

### Collision disassembly produces 80S monosome complexes

The collision stress datasets revealed a previously undescribed 80S conformation comprising ∼5% of ribosomes after 1 h and ∼20% at 4 h (3,573 particles, 9 Å, Figure 2G). This state features a tRNA whose acceptor stem is located in the 60S P-site and attached to nascent chain density visible in the 60S peptide tunnel. Although the associated 40S displays partial density for mRNA, it is not base-paired with the anticodon loop of the P-tRNA, which is ∼23 Å away (Figure 2H). These features suggest that this state, which we term OFF P-tRNA, is not an active translation intermediate, consistent with the absence of neighboring polysome density. Yet, the peptidyl-tRNA and mRNA indicate that it was derived from an elongating ribosome. The simplest explanation is if the OFF P-tRNA state were derived from ASCC-mediated splitting of collided disomes, which would explain why collision stress leads to an increase in 80S monosomes, as seen by polysome profiling. Consistent with this interpretation, stalling stress induced with high-dose ANS showed neither an increased monosome peak on polysome profiles nor the OFF-P tRNA state in cryo-ET analysis (70 μM, Figures S3G–S3I).

To directly test whether OFF P-tRNA ribosomes are a product of collision splitting events, we compared U2OS cells containing or lacking ZNF598,^9^ which is required for ASCC-mediated collision splitting.^14^^,^^15^^,^^16^ Polysome profiling and cryo-ET of wild-type (WT) U2OS cells experiencing collision stress with low-dose ANS revealed increased 80S monosomes and ∼37% of ribosomes in the OFF-P tRNA state, similar to observations in MEF cells. By contrast, ZNF598 knockout (KO) cells in the same stress conditions lacked both a monosomal peak in polysome profiles and the OFF-P tRNA state in cryo-ET image classification (Figures S3J–S3L). Together, these data demonstrate that the OFF-P tRNA monosome species is downstream of collision sensing by ZNF598, likely due to subsequent ASCC-mediated disome dissociation. Reconstitution studies show that ASCC dissociates the lead ribosome of a collision to generate substrates for RQC, so the observed OFF-P tRNA 80S monosome probably represents the final trailing ribosome of a collision pair or queue. This would be consistent with the presence of mRNA in the 40S subunit. High-level accumulation of this product suggests that its further splitting into subunits to allow access by RQC is rate-limiting, presumably because mRNA in the 40S A site impedes access by the Pelota-Hbs1L ribosome rescue complex.^24^

### Collision complexes on compressed polysomes are structurally diverse

**Figure 3:**
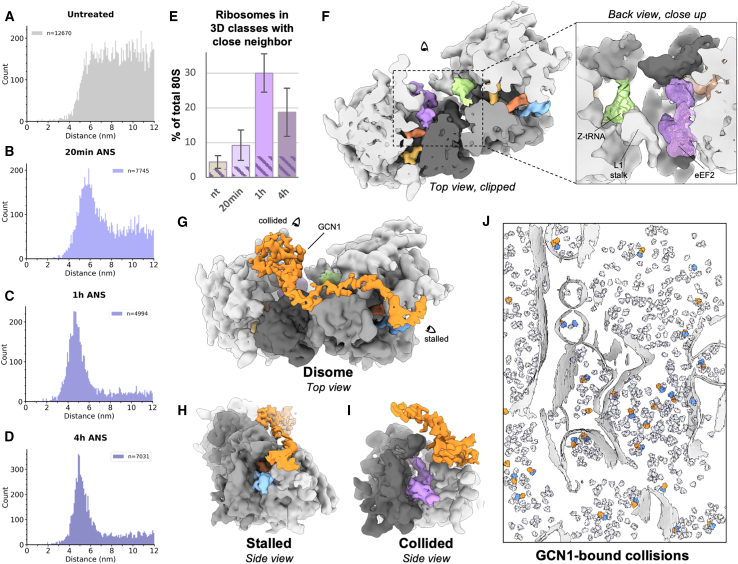
*In situ* analysis of ribosome collisions (A–D) (A) Distance to nearest-neighbor plot for the control dataset, (B) same at 20 min low-dose ANS stress, (C) same at 1 h low-dose ANS stress, and (D) same at 4 h low-dose ANS stress. On each plot, n indicates the number of distances counted in the plot, i.e., all entry/exit distances < 12 nm. (E) Rough quantification of ribosomes with a defined close neighbor based on RELION 3D classification results. Patterned region indicates the proportion of these ribosomes in collisions bound by GCN1 for each condition. Bar and whiskers are mean and SD across tomograms (untreated n = 87, 20 min ANS n = 68, 1 h ANS n = 36, 4 h ANS n = 45). (F) *In situ* subtomogram average of collided disome. Close-up view displays fitted models for the Z-site tRNA and eEF2. (G–J) (G) *In situ* subtomogram average of GCN1-bound collided disomes and side views: (H) of the stalled ribosome, (I) on the collided ribosome. (J) GCN1-bound collisions mapped back into a tomogram. Segmented membranes are displayed in light gray and all other 80S particles in transparent light mauve. See also Figure S4.

To assess the effect of collision stress on the spatial relationships between ribosomes, we conducted nearest-neighbor analysis. For each 80S mRNA entry and exit site, we determined the closest exit and entry sites, respectively, on another 80S within 12 nm (compatible with neighbor particles on the same polysome). In untreated cells, distances varied widely, peaking at ∼7 nm (Figure 3A). However, collision stress led to a gradual compression of these distances over time (Figures 3A–3D), with plots showing a sharp peak at ∼4.5 nm after 1 and 4 h of stress. This distance matches the mRNA length between entry and exit sites in cryoelectron microscopy (cryo-EM) reconstructions of isolated collided disomes.^1^^,^^2^^,^^3^

To visualize the ribosome populations with close neighbors, we used image classification with a mask on neighboring 40S subunits (Figure S4A). We detected ribosomes with defined densities for contacting neighbors in all datasets, including in non-stressed cells, though at varying levels (Figures 3E and S4). In untreated cells, ∼4.4% of 80S ribosomes had close neighbors, increasing to ∼9.2% at 20 min of collision stress, peaking at ∼30% after 1 h, and then decreasing to ∼19% after 4 h. These counts are approximate due to image classification limitations, explaining why ribosomes from these classes plotted back into their original tomograms occasionally show imperfect pairing in Figures S9 and S10. Nonetheless, the decrease in collisions at 4 h is genuine, perhaps due to negative feedback mechanisms that reduce initiation on mRNAs containing collided ribosomes (Figure 1D).^7^^,^^8^ Conversely, U2OS cells lacking ZNF598 had approximately 61% of ribosomes with close neighbors, highlighting the importance of this pathway in limiting the accumulation of collisions in cells (Figure S4B).

To assess ribosome elongation states in collided disomes, we used image classification with a mask around tRNA sites and the GTPase activation center (GAC). Our analysis reveals greater diversity in collisions than previously anticipated from studies on purified disomes.^2^ We identified two lead ribosomes subpopulations: a decoding-like state and a PRE+-like state (Figures S4A–S4C). The density at the stalled ribosome GAC in the decoding class differs from typical eEF1A and likely features a factor involved in the recognition of stalled ribosomes, like DRG2 (Rbg2 in yeast).^6^ Interestingly, the proportion of lead ribosome in the PRE+ conformation increases from ∼11% in untreated cells to 36% at 4 h of collision stress. This suggests that the lead ribosome in a collision is initially found in the decoding Z conformation bound by detection factors and, if left unresolved, may eventually transition to the PRE+ fully stalled state. Similarly, the collided ribosome is also found in two states: a decoding-like state and the rotated-2+ conformation. The rotated-2+ population increases from 65% in untreated cells to 90% after 4 h, suggesting that it too may undergo a conformational transition over time if the collision is not resolved promptly (Figures S4A–S4C).

Despite this heterogeneity, the most abundant disome class in our data displayed an overall geometry similar to purified *XBP1u*-stalled human disomes (Figure 3F),^2^ but with key differences at the inter-ribosome interface. Whereas the *in vitro* structure shows an interface between only the 40S subunits, our *in situ* structure contains an extra Z-site tRNA, with the L1 stalk in an open conformation on the leading 60S subunit facing an additional eEF2 bound to the rotated-2 trailing ribosome (Figure 3F). It is likely that both the Z-tRNA and eEF2 have detached in previous sample preparation steps of mammalian collision complexes.^1^^,^^2^ Finally, collisions between endoplasmic reticulum (ER)-bound ribosomes adopt a similar arrangement as cytosolic ribosomes, apparently relying on a local negative curvature of the fluid ER membrane (Figures S4D–S4F), as had been speculated in earlier work.^1^ These results collectively show that collisions are not homogeneous entities, differ in several ways from those analyzed *in vitro*, and seem to change conformation over time if left unresolved. These findings raise the intriguing possibility that the different conformations may signal different downstream consequences, an idea that remains to be explored.

### GCN1 binding distinguishes collided disomes from compact helical polysomes

To analyze collision detection factors *in situ*, we performed image classification on stalled and collided ribosome populations, using masks around weak densities. Our approach visualized mammalian GCN1-bound collisions *in situ* resembling purified GCN1-bound yeast disomes (Figures 3G–3J).^6^ A density at the EDF1/Mbf1 binding site is also visible in these collisions (Figures S4G and S4H), in line with collision-dependent EDF1 accumulation on polysomes (Figure S1H). The proportion of total cellular ribosomes present in GCN1-bound collisions increases from ∼2% in untreated cells to ∼6% at 1 and 4 h of ANS-induced collision stress (Figure 3E, dashed regions of histogram). GCN1 is found on the early state of collided complexes containing stalled decoding-like ribosomes rather than the later PRE-like stalled state (Figure S4A). This observation provides *in vivo* support for the previous proposal that GCN1 is an early checkpoint for ribosome collisions.^6^

Our analysis also revealed a class of ribosomes with trailing and leading neighbors at a similar distance, but less defined and with an angle distinct from that observed in reconstructions of purified collided disomes (Figures S4I and S4J). This class corresponds to a compact helical polysomal arrangement, one of several arrangements described in earlier cryo-ET analyses of untreated cells.^30^ In our dataset, the helical polysomes mostly contain ribosomes in a POSTi translocation intermediate step, indicating that they can initiate translocation, as opposed to collided rotated-2 ribosomes. The helical polysome class is found at all collision stress time points (Figures S4A–S4C), but not in untreated MEF cells, suggesting that it arises from acute collisions that have yet to be resolved. Consistent with this idea, the helical configuration is especially prominent in polysomes generated by *in vitro* translation on a truncated mRNA.^31^ It was also observed on non-truncated mRNA in long-term *in vitro* translation reactions,^32^ a system that is more prone to stalling than in cells.^1^

Extrapolation of the typical collided disome arrangement shows a maximal polysome length of 4 ribosomes before clashes occur, whereas the helical geometry is not limited in polysome length (Figures S4K and S4L). This suggests that the helical configuration might be more prominent under conditions where collision resolution is slow or saturated. This difference in configuration probably has functional consequences, given that the middle ribosomes within a polysomal helix do not have appreciable GCN1 in any of our cyro-ET datasets. Modeling suggests that the helical arrangement is incompatible with GCN1 binding due to clashes with the P-stalk of the trailing ribosome (Figures S4M–S4O). Together, these results argue that GCN1 specifically recognizes early collided disomes but cannot bind the compact helical polysomes that probably form later downstream unresolved persistent collisions. These observations provide a plausible structural explanation for how different downstream consequences could be triggered in a time-dependent manner in cells experiencing unresolved collision stress.

### Non-functional tRNA-bound 60S particles accumulate under persistent collision stress

**Figure 4:**
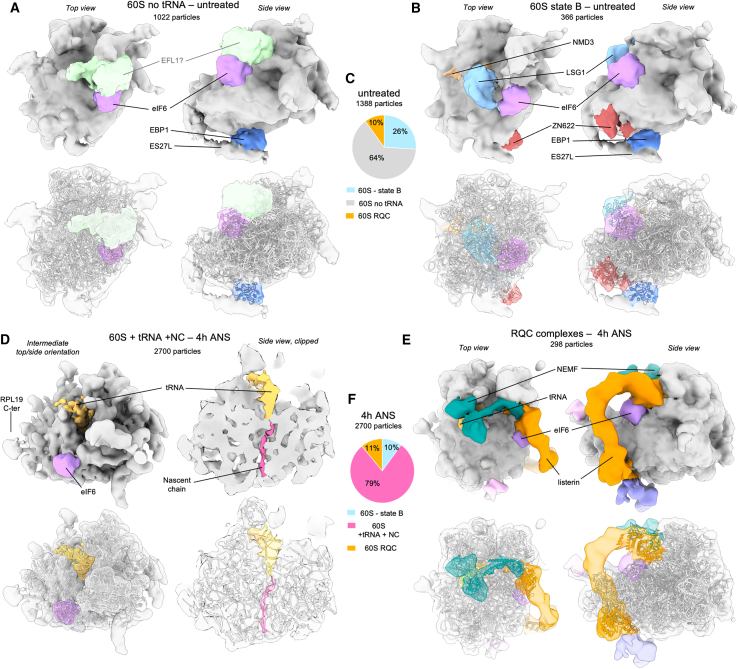
*In situ* subtomogram averages of 60S complexes (A) Major 60S complex observed in control cells displaying densities corresponding to eIF6 (purple), EBP1 (blue), and a putative eFL1 (light green). PDB coordinates for 60S, eIF6, and EBP1 were fitted independently (using the corresponding chains from PDB: 7OW7 for the 60S and eIF6, and PDB: 6LSR for EBP1). (B) Second-most abundant 60S class, corresponding to previously described maturation state B, displaying densities corresponding to eIF6 (purple), LSG1 (light blue), NMD3 (beige), and ZN622 (dark red). Fitted PDB coordinates (PDB: 6LSR). (C) Relative abundances of the 60S complexes observed in the control dataset. (D) Major 60S complex observed in the 4 h low-dose ANS stress dataset displaying densities for eIF6 (purple), partial P-site tRNA (gold), and nascent chain (hot pink). PDB coordinates were fitted independently using the corresponding chains from 3J92 for 60S, eIF6, and tRNA, and 5AJ0 for the nascent chain. (E) NEMF- and Listerin-bound 60S particles, as observed in stressed cells. Fitted PDB coordinates (PDB: 3J92). (F) Relative abundances of the 60S complexes observed in 4 h low-dose ANS stress dataset. See also Figure S5.

The steps downstream of ribosome collisions include the ASCC-mediated splitting of the stalled lead ribosome into 40S and 60S subunits, the latter of which engages RQC. We analyzed RQC by comparing 60S particles in the untreated and 4 h collision stress datasets (with ∼1,500 and ∼2,700 particles, respectively). In untreated cells, the major population (∼64%) featured an idle 60S particle with additional density at RPL23, the GAC, and the exit tunnel (Figures 4A and 4C). Based on their shapes and positions, the RPL23-bound density is probably eIF6, the exit tunnel density is probably EBP1, and the GAC density could be EFL1, which is involved in the release of eIF6 at a late step of ribosome maturation. The other major 60S population (∼26%) strongly resembles the earlier cytosolic maturation intermediate state B (Figures 4B and 4C),^33^ with densities matching LSG1, NMD3, eIF6, ZN622, and EBP1. These assignments suggest that most 60S particles in untreated cells are probably late 60S maturation intermediates, although some may also be 60S intermediates recycled from canonical termination.

After 4 h of collision stress, idle 60S particles were absent and the proportion of state B maturation intermediate decreased to ∼10% (Figure 4F), potentially due to reduced ribosome biogenesis under conditions of reduced protein synthesis (Figure 1D). Instead of these maturation intermediates, most 60S particles (79%) contain densities for a P-site tRNA attached to a nascent chain, eIF6 and EBP1 (Figures 4D, 4F, and S11). The peptidyl-tRNA indicates that these 60S particles are derived from previously translating ribosomes, presumably by disassembly of collision complexes. Peptidyl-tRNA-60S complexes are recognized by the RQC nuclear export mediator Factor (NEMF) to facilitate recruitment of the E3-ligase Listerin for the ubiquitination of the truncated nascent chain.^18^^,^^19^ Subsequent classification revealed densities typical for NEMF and Listerin^19^ on a class comprising ∼10% of the total 60S in both the untreated and 4 h ANS datasets. Additional tRNA-bound populations, possibly bound by NEMF alone, were observed in the 4 h ANS conditions (Figure S5). These findings suggest that untreated cells continually access RQC (consistent with low-level collisions), that only a modest amount of additional NEMF exists to handle elevated collision stress, and that Listerin is the most limiting factor in resolving post-splitting peptidyl-tRNA-60S complexes. Our finding that peptidyl-tRNA-associated 60S subunits accumulate after collision stress is consistent with an early study showing 60S subunit accumulation after similar low-dose ANS treatment.^34^ At the time, this observation was ascribed to a failure of 60S subunit joining, although the reason was unknown. It now emerges that saturation of RQC during collision stress leads to non-empty 60S subunits that cannot participate in translation.

### Collision stress induces progressive impairment of translation initiation

Partial protein synthesis inhibition (by ∼2-fold) in MEF cells exposed to 200 nm ANS is associated with reduced eIF2α phosphorylation, potentially to promote a compensatory increase in translation initiation. These same conditions also lead to a range of stalled, elongating, and collided states, multiple signaling pathways downstream of collisions, and increased recycling of stalled 80S ribosomes into 40S and 60S subunits. To understand the consequences of this multi-tiered perturbation for translation initiation, we analyzed the molecular compositions of initiation complexes. Because these populations are less abundant than 80S populations, quantifications are less precise; therefore, we focused our analysis only on the major differences between normal and collision stress conditions, using cells with acute arsenite-induced ISR as a control for inhibition of initiation.

**Figure 5:**
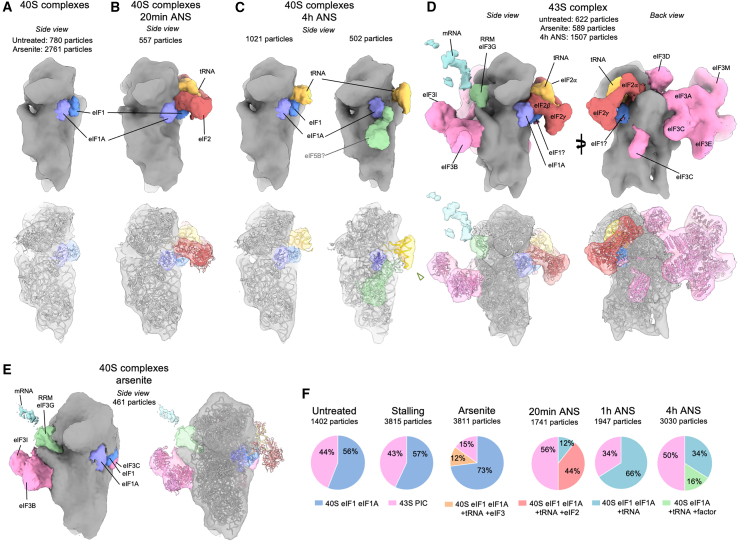
*In situ* subtomogram averages of 40S complexes (A) 40S complex observed in control cells, densities corresponding to eIF1 and eIF1A are displayed in blue and purple, respectively. Fitted PDB coordinates (PDB: 4KZY). (B) Abundant 40S complex appearing at 20 min low-dose ANS stress, displaying extra densities corresponding to tRNA (gold) and eIF2 (red). (C) Major 40S complexes observed at 4 h low-dose ANS stress displaying a P-site tRNA (gold) and a density depicted in light green, possibly fitting eIF5B (PDB coordinates [PDB: 7TQL]). (D) 43S complexes observed in all datasets, displaying eIF3 (pink) and an extra density shown in light cyan proposed to correspond to an mRNA. Fitted PDB coordinates (PDB: 6ZMW). (E) Subtomogram average of a 40S complex with eIF3 but lacking eIF2 and tRNA, in cells treated with 500 μM arsenite for 15 min. Fitted PDB coordinates (PDB: 6ZMW). (F) Relative abundances of the 40S complexes observed in untreated cells and under high-dose ANS stalling, arsenite, or low-dose ANS stress. See also Figure S6.

All datasets contained 40S and 43S complexes (Figures 5A–5F), both of which differed between untreated and collision stress conditions, although they were not affected by stalling stress. Untreated cells showed 40S subunits with densities that match eIF1 and eIF1A (Figure 5A), consistent with a pre-43S assembly state.^35^ This complex remained the most abundant class, even after induction of the ISR with arsenite, consistent with the ISR acting at a later step in initiation. In contrast to these two control conditions, acute collision stress for 20 min led to an abundant 40S complex with bound eIF1, eIF1A, eIF2, and P-tRNA, but lacking eIF3 (Figure 5B). At 4 h of collision stress, we find 2 other 40S populations (Figure 5C). The first contains eIF1 and eIF1A, with an additional tRNA, while the second carries eIF1A, a differently positioned tRNA, and an extra factor. Based on its shape and position, this factor is provisionally ascribed to eIF5B and a de-acylated tRNA (Figure 5C).^36^^,^^37^ These collision-specific aberrant populations may represent 40S complexes that failed to accomplish subunit joining due to insufficient free 60S complexes. Consistent with this interpretation, most 60S complexes in cells experiencing collision stress are occupied with peptidyl-tRNA. Thus, a high rate of collision combined with saturated RQC activity may lead to aberrant initiation complexes arising from 60S insufficiency.

43S complexes represent the well-studied preinitiation complex (PIC) featuring densities matching eIF1, eIF1A, eIF2α, eIF2β, eIF2γ, eIF3, eIF4A1, and a P-site t-RNA (Figure 5D).^38^^,^^39^^,^^40^^,^^41^ In untreated cells where translation is highly active, 44% of particles were 43S PICs. Arsenite-induced ISR activation led to a marked reduction in PICs to 15% and the formation of 43S PICs lacking eIF2 and P-tRNA, as expected for cells that have high levels of eIF2α phosphorylation. By contrast, collision stress showed relatively modest changes in the proportion of 43S complexes, which matches the observation that eIF2α phosphorylation is unchanged or even reduced. A subclass in the 4 h collision stress dataset (551 particles) and the stalling dataset (307 particles) displayed 43S complexes with an extra density compatible with eIF4G and eIF4A in size and shape (Figure S6A).^40^ These are subunits of the 4F complex necessary for scanning, so this class may represent 43S particles that have not yet reached the start codon. It is unclear why this complex seems to be more prominent in cells experiencing persistent collision stress.

The other difference between 43S complexes from untreated cells and cells experiencing collision stress was a strong but ill-defined density at the mRNA entry channel in untreated cells that progressively weakened with increasing time of collision stress (Figures S6C–S6F). Flexible density in this position is consistent with mRNA, which we postulated might be preferentially stabilized in untreated or stalled cells by an adjacent 80S complex that had just initiated elongation (Figures S6G–S6J). This hypothesis was supported by nearest-neighbor analysis, which showed an inverse correlation between the distance to an 80S complex and strength of this density across our conditions (Figures S6K–S6N). These results indicate that the frequency of 43S complex arrival relative to 80S departure from the start codon decreases under collision stress conditions. The reason for this altered timing might be due to reduced overall efficiency of initiation during persistent collision stress.

### Conclusions and perspective

The ability to directly visualize biochemical pathways and reactions in their native context affords an important complement to their analyses in lysates or purified reconstituted systems. First, all the components are at their endogenous concentrations and locations. Second, labile interactions that might be lost in biochemical sample processing can be preserved. Third, the spatial relationships between different components and their positions relative to other cellular structures can be determined. Fourth, multiple biological processes can be evaluated from the same sample and dataset rather than devising separate bespoke assays for different reactions. Finally, the reactions occur at physiologic pH, salt, metabolite, and crowding conditions that are difficult to recapitulate *in vitro*. We have leveraged these advantages to analyze mammalian translation and quality control *in situ* and determine how key constituents of these pathways change with persistent collision stress.

A number of insights emerge from our work, generating hypotheses and directions for future work (Figure S18). First, we visualized tRNA bound to the Z site of elongating ribosomes that resemble the PRE and decoding states. The data suggest that Z-site tRNA is favored by transient deceleration or persistent stalling of a ribosome, possibly due to inefficient ejection of the deacylated tRNA, and is therefore more common in cells undergoing collision stress.

Second, the interface between collided ribosomes extends beyond small subunits and involves a Z-site tRNA and open L1 stalk on the stalled ribosome and eEF2 on the collided rotated-2 neighbor. This larger and more constrained interface would disfavor head swivel motion and eEF2 release on the collided ribosome, which might explain why translocation is impaired.

Third, the collided disome is not a homogeneous unit, as previously thought, but varies in its conformation and composition over time. The important implication of this finding is that different collided states may encode information about the severity, time, or source of stalling, allowing the cell to respond in different ways. Indeed, the ISR effector GCN1 associates with early transient collisions, included in untreated cells, but not with helical polysomes, which only accumulate under acute collision situations. It is attractive to speculate that another factor, such as ZAKα, could recognize static helical ribosomes and initiate a different downstream signaling program.

Fourth, ASCC-mediated collision resolution leaves behind a previously unappreciated OFF-P tRNA 80S monosome that seems to be inefficiently recycled.

Fifth, saturation of the RQC during persistent collision stress leads to the accumulation of peptidyl-tRNA-60S complexes at the expense of free 60S available for subunit joining.

Sixth, a substantial proportion of initiation complexes in cells experiencing persistent collision stress are aberrant, possibly as a consequence of free 60S insufficiency.

Each of these insights highlights the value and power of cryo-ET for integrating native conformational and spatial information in cells to discriminate between previously proposed models and generate hypotheses for future work. Our approach represents a blueprint for future cellular cryo-ET studies of the translation machinery in various physiological and pathological conditions.

### Limitations of the study

This study does not address the functional relevance of all observations revealed by our approach. This is largely due to the lack of methods thus far to target specific ribosomal conformations for isolation or in cell functional assays. For instance, the exact functional implications of the Z-site tRNA remain to be tested, but methods to do so must first be developed. It is nearly impossible to manipulate essential components such as the L1 stalk or eEF2, as numerous secondary consequences would make any observations uninterpretable. Future molecular dynamics studies could be informative to compare the dynamics of a tRNA bound to the Z site or E site, as well as to explore differences in the presence or absence of a collided ribosome. Our results using ZNF598 KO cells suggest that OFF-P tRNA ribosomes arise downstream of disome ubiquitination and splitting, but we cannot fully exclude the possibility that they are generated via a different function of ZNF598. In addition, our work does not address the potential impact of these ribosomes on cells or their recycling mechanism(s). Our data further indicate that initiation is impaired during persistent collision stress, but functional assays that measure the existence and consequences of futile initiation events in cells remain to be developed. Finally, the extent to which our conclusions based on ANS-induced collision stress apply to other triggers of ribosome collisions remains to be investigated experimentally.

## STAR★Methods

### Key resources table

**Table:** 

REAGENT or RESOURCE	SOURCE	IDENTIFIER
**Antibodies**		

Total eIF2α	Cell Signaling	#9722; RRID: AB_2230924
phospho-eIF2α	Cell Signaling	#9721; RRID: AB_330951
phospho-p38	Cell Signaling	#4511; RRID: AB_2139682
phospho-JNK1+2	Cell Signaling	#4668; RRID: AB_823588
PARP-1	Cell Signaling	#9542; RRID: AB_2160739
Caspase 3	Cell Signaling	#9662; RRID: AB_331439
GCN1L1	Thermo Fisher Scientific	A301-843A-M; RRID: AB_1264319
EDF1	Abcam	#ab174651; RRID: AB_2893192
ZNF598	Thermo Fisher Scientific	A305-108A-M; RRID: AB_2782415
RPL35	Thermo Fisher Scientific	PA552245; RRID: AB_2646744
RPS10	Abcam	#ab151550; RRID: AB_2714147

**Chemicals, peptides, and recombinant proteins**		

Anisomycin	Sigma-Aldrich	A9789
Arsenite	Supelco	1062771000
35S-methionine label	Hartmann Analytic	ARS0110
Cycloheximide	Sigma-Aldrich	C7698
RNAsin Ribonuclease inhibitor	Promega	N2515
Complete EDTA-free protease inhibitor	Roche	5056489001
Phosphatase inhibitor cocktail 100x	Cell Signaling	#5870

**Deposited data**		

In situ subtomogram average densities of ribosomal complexes in collision stress and control cells	This study	EMDB: 19211, 19212, 19213, 19214, 19215, 19216, 19217, 19218, 19219, 19220, 19221, 19222, 19223, 19224, 19225, 19226, 19227, 19228, 19229, 19230, 19231, 19232, 19233, 19234, 19235, 19236, 19237, 19238, 19239, 19240, 19241
Code for ribosome nearest neighbor distance analysis	This study	https://www.doi.org/10.5281/zenodo.10475883

**Experimental models: Cell lines**		

Mouse MEF cells	M. Molinari lab	N/A
U2OS wt cells	S. Bekker-Jensen lab	N/A
U2OS ZNF598 KO	S. Bekker-Jensen lab	N/A

**Software and algorithms**		

SerialEM	Mastronarde et al.^42^	https://bio3d.colorado.edu/SerialEM/
Warp	Tegunov and Cramer^43^	http://www.warpem.com/warp/?page_id = 65
IMOD	Mastronarde and Held^44^	http://bio3d.colorado.edu/imod/
pyTom	Hrabe et al.^45^ Chaillet et al.^46^	https://github.com/SBC-Utrecht/pytom-template-matching-gpu
Relion version 3.1	Scheres^47^	https://www3.mrc-lmb.cam.ac.uk/relion/index.php/Main_Page
M	Tegunov et al.^48^	http://www.warpem.com/warp/?page_id = 65
Tomosegmemtv	Martinez-Sanchez et al.^49^	https://sites.google.com/site/3demimageprocessing/tomosegmemtv
UCSF ChimeraX	Pettersen et al.^50^	https://www.cgl.ucsf.edu/chimerax/
ArtiaX plugin	Ermel et al.^51^	https://github.com/FrangakisLab/ArtiaX

### Resource availability

#### Lead contact

Further information and requests for resources and reagents should be directed to and will be fulfilled by the lead contact, Juliette Fedry (jfedry@mrc-lmb.cam.ac.uk).

#### Materials availability

This study did not generate new unique reagents.

#### Data and code availability

• Electron density maps are deposited in the Electron Microscopy Data Bank with the following accession numbers: 19211, 19212, 19213, 19214, 19215, 19216, 19217, 19218, 19219, 19220, 19221, 19222, 19223, 19224, 19225, 19226, 19227, 19228, 19229, 19230, 19231, 19232, 19233, 19234, 19235, 19236, 19237, 19238, 19239, 19240, 19241.
• The Python script used for nearest neighbor analysis is deposited at Github: https://github.com/mvanevic/polysome_mef and the corresponding doi is: https://www.doi.org/10.5281/zenodo.10475883
• Any additional information required to reanalyze the data reported in this paper is available from the lead contact upon request.

### Experimental model and study participant details

#### Cell lines

wt MEF cells were a kind gift from M. Molinari. Wt and ZNF598 KO cells were a kind gift from S. Bekker-Jensen. Cells were grown in DMEM with 10% Fetal Bovine Serum (FBS) at 37°C and 5% CO_2_. Prior to experiments cells were treated as indicated: with 200 nM ANS (collisions stress) for 15min, 1h or 4h, or with 70 μM ANS for 3h30 (stalling stress), or with 500 μM arsenite for 10-15min.

### Method details

#### Annexin V / PI staining

Cells were stained using an Annexin V / PI apoptosis detection kit (Biolegends) according to the manufacturer’s instructions and analyzed by FACS.

#### Western blotting antibodies

The cell surface was briefly washed with PBS and the cells were harvested in trypsin (+ANS for stressed conditions). Cells were washed once in ice cold PBS and pellets were snap frozen until further use. Cell lysates were prepared by solubilization of the cell pellet in TEN Triton buffer (20mM Tris pH 7.5, 150mM NaCla, 1mM EDTA), supplemented with Protease Inhibitor (Roche) and phosphatase inhibitor (Cell Signaling #5870), at 4°C for 30min. Cell lysates were centrifuged at 20 000g for 20 min and the supernatant was diluted in 2X sample DTT-loading buffer. Samples were heated to 95 degrees for 5min and stored at -20. For western blotting we used following commercial antibodies: eIF2α (Cell Signaling Antibody #9722), phospho-eIF2α (Cell Signaling #9721), phospho-p38 (Cell Signaling #4511), phospho-JNK1+2 (Cell Signaling mAb #4668), caspase 3 (Cell Signaling #9542), PARP-1 (Cell Signaling #9662), GCN1L1 (Thermo Fisher Scientific A301-843A-M), EDF1 (Abcam #ab174651), ZNF598 (Thermo Fisher Scientific A305-108A-M), RPL35 (Life Technologies PA552245), RPS10 (Abcam #ab151550). Relative band intensity quantification was performed in Fiji/ImageJ.

#### S35 protein synthesis

MEF cells were grown in DMEM FBS and treated with 100μg/mL CHX or 200nM ANS for 15min, 1h, or 4h prior to labelling. Cells were then treated with DMEM methionine-free media (ThermoFisher Scientific #21013024) for 20 min and incubated with 30 μCi/ml 35S-methionine label (Hartmann Analytic) for 1 h. After washing the samples with PBS, proteins were extracted with lysis buffer (50 mM TrisHCl pH 7.5, 150 mM NaCl, 1% Tween-20, 0.5% NP-40, 1× protease inhibitor cocktail (Roche) and phosphatase inhibitor cocktail (Sigma Aldrich) and precipitated onto filter paper (Whatmann) with 25% trichloroacetic acid and washed twice with 70% ethanol and twice with acetone. Scintillation was then read using a liquid scintillation counter (Perkin Elmer) and the activity was normalized by total protein content. All experiments were done in technical triplicates for each biological unit.

#### Polysome profiling

MEF cells were grown to about 50-70% confluency and harvested with trypsin (+ANS in the stress conditions). Cells were washed once in ice cold PBS (+ANS) prior to lysis for 30min on ice (lysis buffer: 50mM Hepes KOH pH7.4, 15mM MgOAc, 100mM KOAc, 5% glycerol, 1% triton X-100, 0.5% sodium deoxycholate, 1mM DTT, 1mM PMSF, 6U/mL RNAsin, protease inhibitor tablet). Lysate was cleared by centrifugation at 8000 g for 5 min and added on top of a 10-50% sucrose gradient (in buffer: 25mM Hepes-KOH, 100mM KOAc, 5mM MgOAc, 1mM DTT, 500 μg/mL heparin, 1mM PMSF). Gradients were centrifuged for 3h30 at 32 000 rpm in a 32.1 Ti rotor (Beckman). Gradients were sampled and OD was measured using a gradient station (Biocomp).

#### Grid preparation

MEF were seeded on R2/2 holey carbon on gold grids (Quantifoil or Protochips) coated with fibronectin in a glass bottom dish (Mattek or Ibidi) and incubated for ∼24 h. For stress conditions, cells were then incubated with 200 nM anisomycin in DMEM + 10% FBS for 20min, 1h or 4h. Grids were incubated for 5min in DMEM (+ 200 nM ANS for stressed conditions) with 10% dextran-40 used as non cell permeable cryo-protectant.^52^ Grids were then immediately mounted to a manual plunger, blotted from the back for ∼10 s and plunged into liquid ethane.

#### Lamella preparation

Lamellae were prepared using an Aquilos FIB-SEM system (Thermo Fisher Scientific) based on Rigort et al.^53^ and equipped with a CERES Ice shield (Delmic). Grids were sputtered with an initial platinum coat (10 s) followed by a 10 s gas injection system (GIS) to add an extra protective layer of organometallic platinum. Samples were tilted to an angle of 15° to 22° and 10 μm wide lamellae were prepared. The milling process was performed with an ion beam of 30 kV energy in 3 steps: 1) 500 pA, gap 3 μm with expansion joints, 2) 300 pA, gap 1 μm, 3) 100 pA, gap 500 nm. Lamellae were finally polished at 30-50 pA with a gap of 200 nm.

#### Data collection for non-treated, 20min, 1h ANS, datasets

A total of respectively 93 (untreated), 74 (20 min ANS), 45 (1h ANS), 199 (stalling), 148 (U2OS 4h ANS), 168 (U2OS ZNF598KO 4h ANS) and 123 (arsenite) tilt series were acquired on a Talos Arctica (Thermo Fisher Scientific) operated at an acceleration voltage of 200 kV and equipped with a K2 summit direct electron detector and 20eV slit energy filter (Gatan). Images were recorded in movies of 5-8 frames at a target defocus of 4 to 6 μm and an object pixel size of 2.17 Å. Tilt series were acquired in SerialEM using a grouped dose-symmetric tilt scheme covering a range of ±54° with a pre tilt of ±10° and an angular increment of 3°. The cumulative dose of a series did not exceed 80 e-/Å2.

#### Data collection for 4h ANS dataset

A total of 53 tilt series were acquired on a Titan Krios (Thermo Fisher Scientific) equipped with a K3 summit direct electron detector and Bioquantum energy filter (Gatan). The microscope was operated at an acceleration voltage of 300 kV and 20eV slit. Images were recorded in movies of 10 frames at a target defocus of 4 to 6 μm and an object pixel size of 2.17 Å. Tilt series were acquired in SerialEM^42^ using a grouped dose-symmetric tilt scheme covering a range of ±54° with a pre tilt of ±10° and an angular increment of 3°.^54^ The cumulative dose of a series did not exceed 80 e-/Å2.

#### Tomogram reconstruction

The cryoET data processing workflow is represented in Figure S2. Movie files of individual projection images were motion- and CTF-corrected in Warp and combined into stacks of tilt series.^43^ The combined stacks were aligned using patch tracking in IMOD.^44^ CTF estimation for entire tilt series was performed in Warp^43^ and full tomograms were reconstructed by weighted back projection at a pixel size of 17.36 Å. Ice thickness was determined manually and was found to be <200nm for all lamellae.

#### Particle picking

Particle coordinates were determined using PyTom^45^^,^^46^ template matching against a reconstruction of a mammalian 80S, 40S or 60S ribosomes, filtered to 40 Å. The determined positions of ribosomes were used to extract subtomograms and corresponding CTF volumes at a pixel size of 8.68 Å (4× binned) in Warp^43^ for the 80S ribosomes and 4.34 Å (2× binned) for the 40S and 60S particles.

#### Subtomogram analysis

The extracted subtomograms were used for 3D classification with image alignment against a low pass filtered 80S/60S/40S ribosome map as reference in RELION (3.1.4)^47^ to exclude false positives. The remaining ribosome subtomograms were refined in RELION and good 80S particles were re-extracted in Warp at a pixel size of 4.34 Å (2x binned). Bin2 80S subtomograms were refined in RELION with a mask on the LSU prior to a first round of 3D classification without image alignment with a mask on the SSU to separate rotated from non-rotated ribosomes. A second round of classification was performed using a mask positioned on the tRNA and elongation factors sites, optimizing the mask extension and class number to yield stable classes. The classes containing a high number of particles (decoding E) were further submitted to 3D classification without alignment with 6-10 classes to analyze the presence of smaller sub-populations (like POST and POSTi). The different classes were finally subjected to an iterative refinement in M.^48^

Bin2 40S and 60S subtomograms were submitted to 3D refinement in RELION followed by rounds of 3D classification without image alignment, introducing masks on densities visible at low threshold, in order to separate the corresponding particles.

#### Spatial analysis

For each 80S ribosome particle, the position and angles from RELION star file was used to calculate the position of the mRNA entry and exit sites and distances were calculated from its mRNA entry site to all other particles mRNA exit sites; the shortest distance was retained. Conversely, the shortest distance of all other entry sites to a particle exit site was also computed. The distribution of these shortest distances was plotted using matplotlib.

#### Visualization

Membranes were segmented using Tomosegmemtv.^49^ All figures were prepared in UCSF ChimeraX^50^ using the ArtiaX plugin^51^ for mapping back subtomogram averages to their coordinates in the original tomogram.

#### Quantification and statistical analysis

Western blot relative band intensity quantification was performed in Fiji/ImageJ and plotted Figure 1C. Estimation of relative abundances for the different ribosome populations (Figures 2, 3, 4, 5, and S2–S5) were obtained from Relion 3.1 3D classification analysis. Bar and whiskers on Figure 2E are mean and s.d. across tomograms.

All reported resolutions are based on the Fourier shell correlation (FSC) 0.143 criterion.^55^

## Supplemental information

Document S1. Figures S1–S6

Document S2. Article plus supplemental information

## References

[1] Juszkiewicz S., Chandrasekaran V., Lin Z., Kraatz S., Ramakrishnan V., Hegde R.S. (2018). ZNF598 Is a Quality Control Sensor of Collided Ribosomes. Mol. Cell.

[2] Narita M., Denk T., Matsuo Y., Sugiyama T., Kikuguchi C., Ito S., Sato N., Suzuki T., Hashimoto S., Machová I. (2022). A distinct mammalian disome collision interface harbors K63-linked polyubiquitination of uS10 to trigger hRQT-mediated subunit dissociation. Nat. Commun..

[3] Ikeuchi K., Tesina P., Matsuo Y., Sugiyama T., Cheng J., Saeki Y., Tanaka K., Becker T., Beckmann R., Inada T. (2019). Collided ribosomes form a unique structural interface to induce Hel2-driven quality control pathways. EMBO J..

[4] Cerullo F., Filbeck S., Patil P.R., Hung H.C., Xu H., Vornberger J., Hofer F.W., Schmitt J., Kramer G., Bukau B. (2022). Bacterial ribosome collision sensing by a MutS DNA repair ATPase paralogue. Nature.

[5] Saito K., Kratzat H., Campbell A., Buschauer R., Burroughs A.M., Berninghausen O., Aravind L., Green R., Beckmann R., Buskirk A.R. (2022). Ribosome collisions induce mRNA cleavage and ribosome rescue in bacteria. Nature.

[6] Pochopien A.A., Beckert B., Kasvandik S., Berninghausen O., Beckmann R., Tenson T., Wilson D.N. (2021). Structure of Gcn1 bound to stalled and colliding 80S ribosomes. Proc. Natl. Acad. Sci. USA.

[7] Sinha N.K., Ordureau A., Best K., Saba J.A., Zinshteyn B., Sundaramoorthy E., Fulzele A., Garshott D.M., Denk T., Thoms M. (2020). EDF1 coordinates cellular responses to ribosome collisions. eLife.

[8] Juszkiewicz S., Slodkowicz G., Lin Z., Freire-Pritchett P., Peak-Chew S.Y., Hegde R.S. (2020). Ribosome collisions trigger cis-acting feedback inhibition of translation initiation. eLife.

[9] Vind A.C., Snieckute G., Blasius M., Tiedje C., Krogh N., Bekker-Jensen D.B., Andersen K.L., Nordgaard C., Tollenaere M.A.X., Lund A.H. (2020). ZAKalpha Recognizes Stalled Ribosomes through Partially Redundant Sensor Domains. Mol. Cell.

[10] Wu C.C., Peterson A., Zinshteyn B., Regot S., Green R. (2020). Ribosome Collisions Trigger General Stress Responses to Regulate Cell Fate. Cell.

[11] Stoneley M., Harvey R.F., Mulroney T.E., Mordue R., Jukes-Jones R., Cain K., Lilley K.S., Sawarkar R., Willis A.E. (2022). Unresolved stalled ribosome complexes restrict cell-cycle progression after genotoxic stress. Mol. Cell.

[12] Garzia A., Jafarnejad S.M., Meyer C., Chapat C., Gogakos T., Morozov P., Amiri M., Shapiro M., Molina H., Tuschl T. (2017). The E3 ubiquitin ligase and RNA-binding protein ZNF598 orchestrates ribosome quality control of premature polyadenylated mRNAs. Nat. Commun..

[13] Sundaramoorthy E., Leonard M., Mak R., Liao J., Fulzele A., Bennett E.J. (2017). ZNF598 and RACK1 Regulate Mammalian Ribosome-Associated Quality Control Function by Mediating Regulatory 40S Ribosomal Ubiquitylation. Mol. Cell.

[14] Hashimoto S., Sugiyama T., Yamazaki R., Nobuta R., Inada T. (2020). Identification of a novel trigger complex that facilitates ribosome-associated quality control in mammalian cells. Sci. Rep..

[15] Juszkiewicz S., Speldewinde S.H., Wan L., Svejstrup J.Q., Hegde R.S. (2020). The ASC-1 Complex Disassembles Collided Ribosomes. Mol. Cell.

[16] Matsuo Y., Tesina P., Nakajima S., Mizuno M., Endo A., Buschauer R., Cheng J., Shounai O., Ikeuchi K., Saeki Y. (2020). RQT complex dissociates ribosomes collided on endogenous RQC substrate SDD1. Nat. Struct. Mol. Biol..

[17] Best K., Ikeuchi K., Kater L., Best D., Musial J., Matsuo Y., Berninghausen O., Becker T., Inada T., Beckmann R. (2023). Structural basis for clearing of ribosome collisions by the RQT complex. Nat. Commun..

[18] Shao S., Hegde R.S. (2014). Reconstitution of a minimal ribosome-associated ubiquitination pathway with purified factors. Mol. Cell.

[19] Shao S., Brown A., Santhanam B., Hegde R.S. (2015). Structure and assembly pathway of the ribosome quality control complex. Mol. Cell.

[20] Behrmann E., Loerke J., Budkevich T.V., Yamamoto K., Schmidt A., Penczek P.A., Vos M.R., Bürger J., Mielke T., Scheerer P. (2015). Structural snapshots of actively translating human ribosomes. Cell.

[21] Gemmer M., Chaillet M.L., van Loenhout J., Cuevas Arenas R., Vismpas D., Gröllers-Mulderij M., Koh F.A., Albanese P., Scheltema R.A., Howes S.C. (2023). Visualization of translation and protein biogenesis at the ER membrane. Nature.

[22] Hoffmann P.C., Kreysing J.P., Khusainov I., Tuijtel M.W., Welsch S., Beck M. (2022). Structures of the eukaryotic ribosome and its translational states in situ. Nat. Commun..

[23] Budkevich T.V., Giesebrecht J., Behrmann E., Loerke J., Ramrath D.J., Mielke T., Ismer J., Hildebrand P.W., Tung C.S., Nierhaus K.H. (2014). Regulation of the mammalian elongation cycle by subunit rolling: a eukaryotic-specific ribosome rearrangement. Cell.

[24] Shao S., Murray J., Brown A., Taunton J., Ramakrishnan V., Hegde R.S. (2016). Decoding Mammalian Ribosome-mRNA States by Translational GTPase Complexes. Cell.

[25] Bhaskar V., Graff-Meyer A., Schenk A.D., Cavadini S., von Loeffelholz O., Natchiar S.K., Artus-Revel C.G., Hotz H.R., Bretones G., Klaholz B.P. (2020). Dynamics of uS19 C-Terminal Tail during the Translation Elongation Cycle in Human Ribosomes. Cell Rep..

[26] Budkevich T., Giesebrecht J., Altman R.B., Munro J.B., Mielke T., Nierhaus K.H., Blanchard S.C., Spahn C.M. (2011). Structure and dynamics of the mammalian ribosomal pretranslocation complex. Mol. Cell.

[27] Brown A., Baird M.R., Yip M.C., Murray J., Shao S. (2018). Structures of translationally inactive mammalian ribosomes. eLife.

[28] Wu C.C., Zinshteyn B., Wehner K.A., Green R. (2019). High-Resolution Ribosome Profiling Defines Discrete Ribosome Elongation States and Translational Regulation during Cellular Stress. Mol. Cell.

[29] Xing H., Taniguchi R., Khusainov I., Kreysing J.P., Welsch S., Turoňová B., Beck M. (2023). Translation dynamics in human cells visualized at high resolution reveal cancer drug action. Science.

[30] Brandt F., Carlson L.A., Hartl F.U., Baumeister W., Grünewald K. (2010). The three-dimensional organization of polyribosomes in intact human cells. Mol. Cell.

[31] Brandt F., Etchells S.A., Ortiz J.O., Elcock A.H., Hartl F.U., Baumeister W. (2009). The native 3D organization of bacterial polysomes. Cell.

[32] Myasnikov A.G., Afonina Z.A., Ménétret J.F., Shirokov V.A., Spirin A.S., Klaholz B.P. (2014). The molecular structure of the left-handed supra-molecular helix of eukaryotic polyribosomes. Nat. Commun..

[33] Liang X., Zuo M.Q., Zhang Y., Li N., Ma C., Dong M.Q., Gao N. (2020). Structural snapshots of human pre-60S ribosomal particles before and after nuclear export. Nat. Commun..

[34] van Venrooij W.J., van Eenbergen J., Janssen A.P. (1977). Effect of anisomycin on the cellular level of native ribosomal subunits. Biochemistry.

[35] Sokabe M., Fraser C.S. (2014). Human eukaryotic initiation factor 2 (eIF2)-GTP-Met-tRNAi ternary complex and eIF3 stabilize the 43 S preinitiation complex. J. Biol. Chem..

[36] Kazan R., Bourgeois G., Lazennec-Schurdevin C., Larquet E., Mechulam Y., Coureux P.D., Schmitt E. (2022). Role of aIF5B in archaeal translation initiation. Nucleic Acids Res..

[37] Lapointe C.P., Grosely R., Sokabe M., Alvarado C., Wang J., Montabana E., Villa N., Shin B.S., Dever T.E., Fraser C.S. (2022). eIF5B and eIF1A reorient initiator tRNA to allow ribosomal subunit joining. Nature.

[38] Hashem Y., des Georges A., Dhote V., Langlois R., Liao H.Y., Grassucci R.A., Hellen C.U., Pestova T.V., Frank J. (2013). Structure of the mammalian ribosomal 43S preinitiation complex bound to the scanning factor DHX29. Cell.

[39] des Georges A., Dhote V., Kuhn L., Hellen C.U., Pestova T.V., Frank J., Hashem Y. (2015). Structure of mammalian eIF3 in the context of the 43S preinitiation complex. Nature.

[40] Brito Querido J., Sokabe M., Kraatz S., Gordiyenko Y., Skehel J.M., Fraser C.S., Ramakrishnan V. (2020). Structure of a human 48S translational initiation complex. Science.

[41] Kratzat H., Mackens-Kiani T., Ameismeier M., Potocnjak M., Cheng J., Dacheux E., Namane A., Berninghausen O., Herzog F., Fromont-Racine M. (2021). A structural inventory of native ribosomal ABCE1-43S pre-initiation complexes. EMBO J..

[42] Mastronarde D.N. (2005). Automated electron microscope tomography using robust prediction of specimen movements. J. Struct. Biol..

[43] Tegunov D., Cramer P. (2019). Real-time cryo-electron microscopy data preprocessing with Warp. Nat. Methods.

[44] Mastronarde D.N., Held S.R. (2017). Automated tilt series alignment and tomographic reconstruction in IMOD. J. Struct. Biol..

[45] Hrabe T., Chen Y., Pfeffer S., Cuellar L.K., Mangold A.V., Förster F. (2012). PyTom: a python-based toolbox for localization of macromolecules in cryo-electron tomograms and subtomogram analysis. J. Struct. Biol..

[46] Chaillet M.L., van der Schot G., Gubins I., Roet S., Veltkamp R.C., Förster F. (2023). Extensive Angular Sampling Enables the Sensitive Localization of Macromolecules in Electron Tomograms. Int. J. Mol. Sci..

[47] Scheres S.H. (2012). RELION: implementation of a Bayesian approach to cryo-EM structure determination. J. Struct. Biol..

[48] Tegunov D., Xue L., Dienemann C., Cramer P., Mahamid J. (2021). Multi-particle cryo-EM refinement with M visualizes ribosome-antibiotic complex at 3.5 Å in cells. Nat. Methods.

[49] Martinez-Sanchez A., Garcia I., Asano S., Lucic V., Fernandez J.J. (2014). Robust membrane detection based on tensor voting for electron tomography. J. Struct. Biol..

[50] Pettersen E.F., Goddard T.D., Huang C.C., Meng E.C., Couch G.S., Croll T.I., Morris J.H., Ferrin T.E. (2021). UCSF ChimeraX: Structure visualization for researchers, educators, and developers. Protein Sci..

[51] Ermel U.H., Arghittu S.M., Frangakis A.S. (2022). ArtiaX: An electron tomography toolbox for the interactive handling of sub-tomograms in UCSF ChimeraX. Protein Sci..

[52] Huebinger J., Han H.M., Hofnagel O., Vetter I.R., Bastiaens P.I., Grabenbauer M. (2016). Direct Measurement of Water States in Cryopreserved Cells Reveals Tolerance toward Ice Crystallization. Biophys. J..

[53] Rigort A., Bäuerlein F.J., Villa E., Eibauer M., Laugks T., Baumeister W., Plitzko J.M. (2012). Focused ion beam micromachining of eukaryotic cells for cryoelectron tomography. Proc. Natl. Acad. Sci. USA.

[54] Hagen W.J.H., Wan W., Briggs J.A.G. (2017). Implementation of a cryo-electron tomography tilt-scheme optimized for high resolution subtomogram averaging. J. Struct. Biol..

[55] Rosenthal P.B., Henderson R. (2003). Optimal determination of particle orientation, absolute hand, and contrast loss in single-particle electron cryomicroscopy. J. Mol. Biol..

