# Supplementary material for: Visualization of translation reorganization upon persistent ribosome collision stress in mammalian cells
Source: Mol Cell. Author manuscript; Available in PMC 2024 Apr 29. (PMC7615912; doi:10.1016/j.molcel.2024.01.015)
Supplement: Document S1. Figures S1–S6 [file EMS194230-supplement-Document_S1__Figures_S1_S6.pdf]

Molecular Cell, Volume *84*

Supplemental information

Visualization of translation reorganization upon persistent ribosome collision stress in mammalian cells

Juliette Fedry, Joana Silva, Mihajlo Vanevic, Stanley Fronik, Yves Mechulam, Emmanuelle Schmitt, Amédée des Georges, William James Faller, and Friedrich Förster

Supplementary Figures

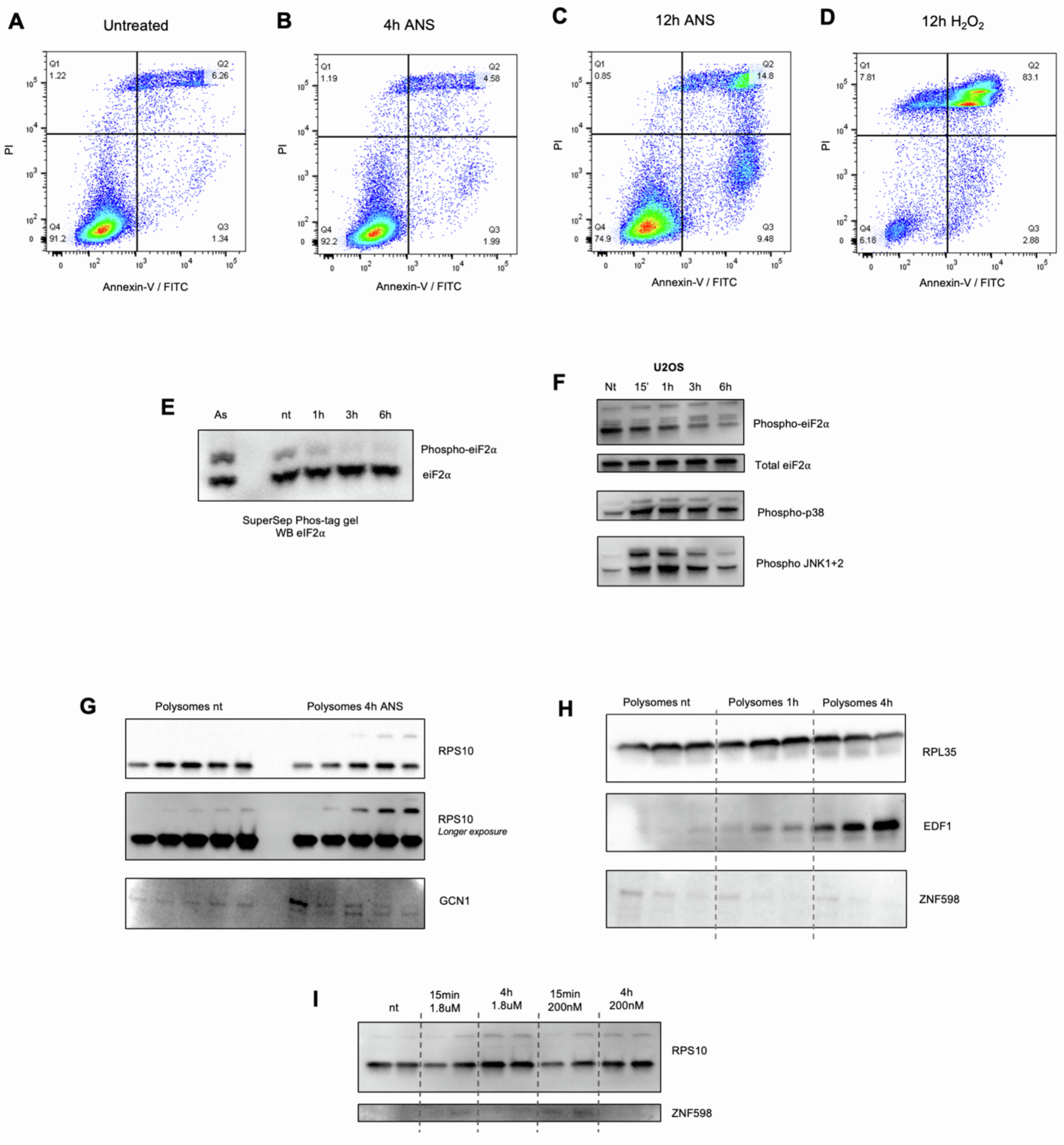

Supplementary Figure S1: FACS and Western Blot analysis of treated cells (related to Figure 1)

Annexin V / PI FACS analysis of cell death in MEF cells (A) untreated, or treated with (B) 4h 200 nM ANS, (C) 12h 200 nM ANS, (D) 12h 1 mM H_2O_2. (E) Analysis of eIF2α phosphorylation in MEF cells on SuperSep Phos-tag gels followed by eIF2α western blot. (F) Analysis of the phosphorylation levels of eIF2α, p38 and JNK in U2OS cells at 15', 1h, 3h and 6h of collision stress (low dose ANS: 200nM). (G) Analysis of the presence of known ribosome collision sensors on polysomes of control (nt) cells and cells stressed with low dose ANS for 1h and 4h. RPL35 is used as loading control antibody, the presence of EDF1 and ZNF598 is tested on polysomes. (H) Detection of RPS10 ubiquitination and GCN1 presence on polysomes of nt and 4h stressed cells. (I) Comparison of RPS10 ubiquitination and ZNF598 detection on polysomes in untreated MEF cells and cells treated with either a low dose (200 nM) or a higher dose (1.8 μM) of ANS for either a short (15 min) or a long (4h) incubation time.

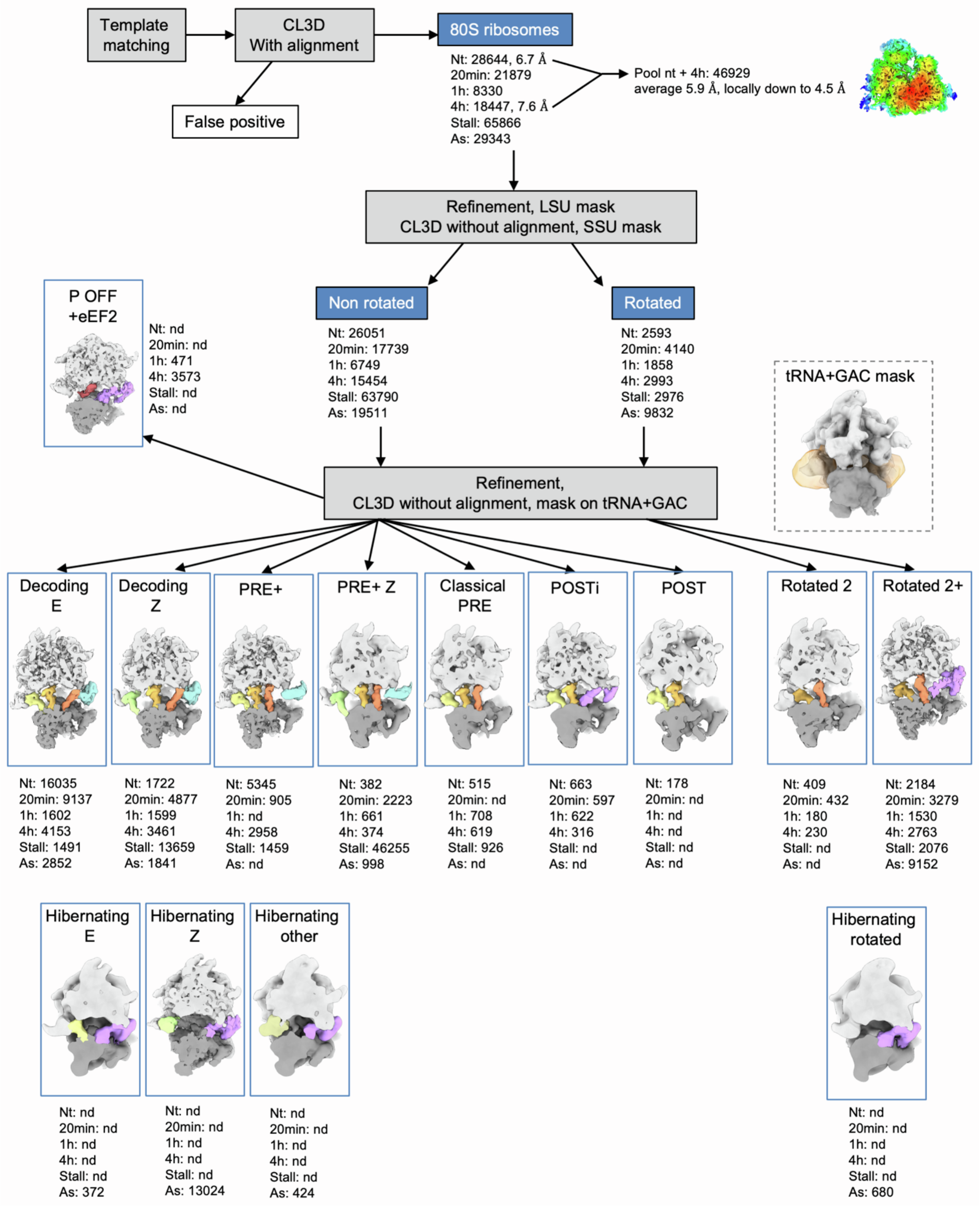

Supplementary Figure S2: Cryo-ET data analysis workflow (related to STAR Methods)

Particles were picked using template matching in PyTom (*60*), subtomograms extracted in Warp (*58*) and false positive were eliminated using CL3D with image alignment in RELION (*61*), followed by subtomogram alignment with a mask on the LSU. The particles were hierarchically classified with CL3D without image alignment, first using a mask on the SSU to separate rotated from unrotated states, and then using a mask on the tRNA sites and the GAC. Final classes were refined in M (*62*).

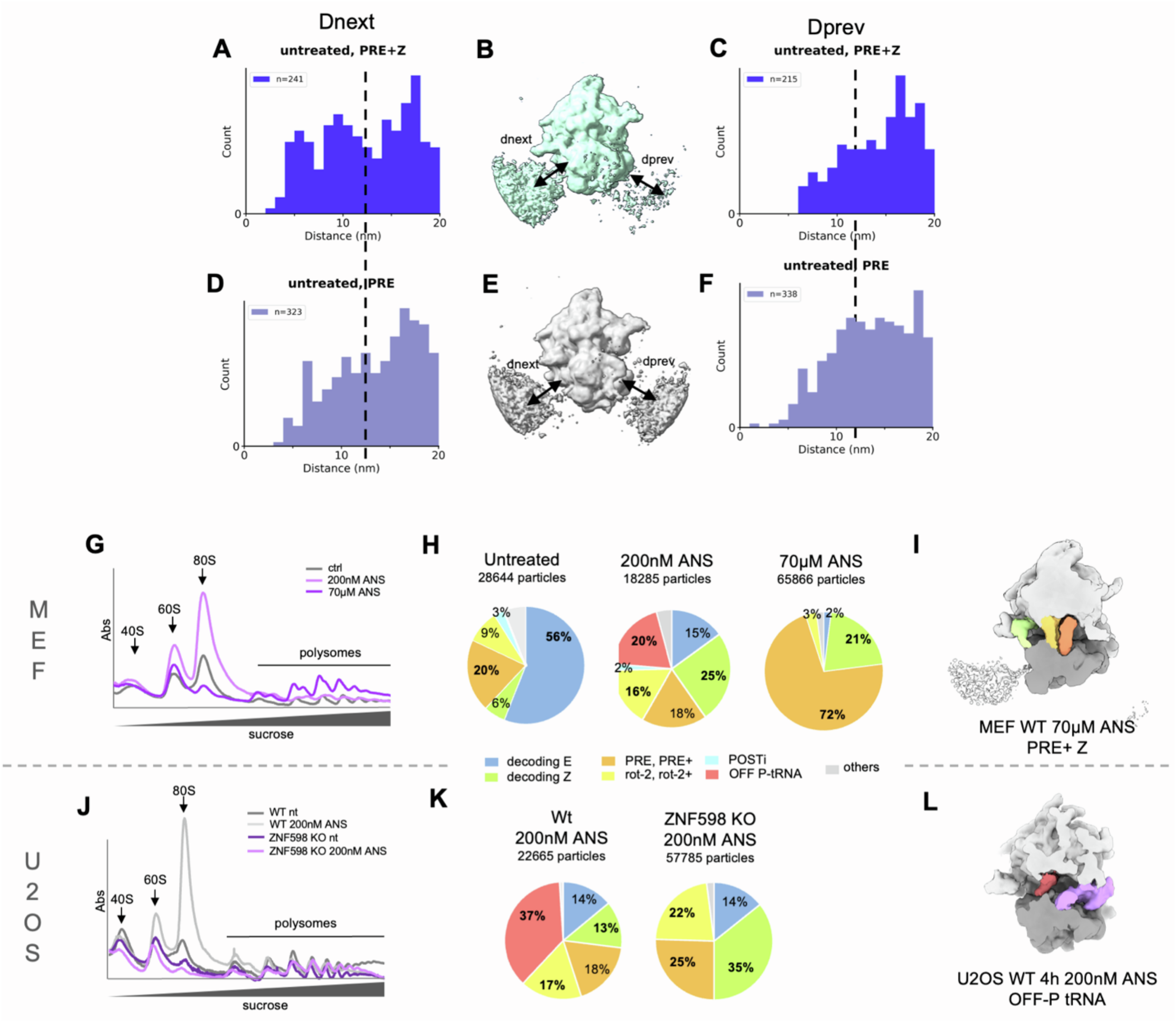

Supplementary Figure S3 (related to Figure 2): Analysis of 80S ribosomal populations associated with translation stress.

Analysis of translation speed of Z-site carrying ribosomes in cells.

(A) Distribution of distances to next neighbor for PRE+ Z ribosomes in the untreated MEF dataset. (B) subtomogram average of PRE+ Z state with stronger density for next neighbor than for the previous one, (C) Distribution of distance to previous neighbor for PRE+ Z ribosomes in the untreated MEF dataset. (D) (E) (F) respectively same as in (A) (B) (C) for PRE ribosomes.

Analysis of ribosomal populations in stalling stress and ZNF598 KO cells.

(G) Polysome profiling on sucrose gradients for MEF cells untreated, and treated with 200 nM or 70 µM ANS. (H) Corresponding relative abundances of 80S complexes determined by cryoET. (**I**) Segmented subtomogram average of the MEF stalled dataset most abundant PRE+ Z class (J) Polysome profiling on sucrose gradients of WT and ZNF598 KO U2OS cells, untreated or treated with 200 nM ANS. (K) Corresponding relative abundances of 80S complexes determined by cryoET. (L) Segmented subtomogram average of the U2OS 4h 200 nM ANS dataset OFF-PtRNA class.

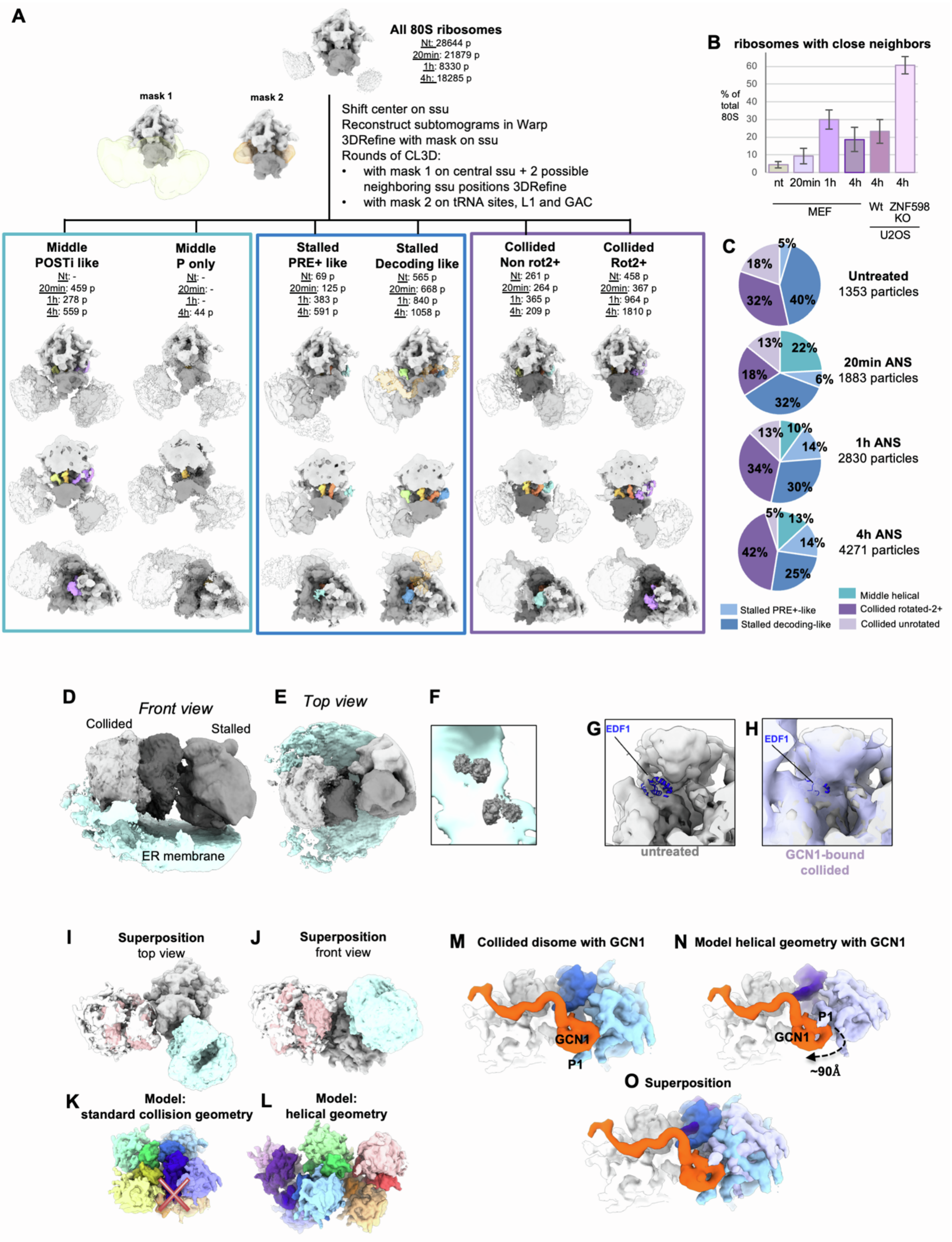
A
All 80S ribosomes
Nt: 28644 p
20min: 21879 p
1h: 8330 p
4h: 18285 p
mask 1
mask 2
Shift center on ssu
Reconstruct subtomograms in Warp
3DRefine with mask on ssu
Rounds of CL3D:
• with mask 1 on central ssu + 2 possible neighboring ssu positions 3DRefine
• with mask 2 on tRNA sites, L1 and GAC
Middle POSTi like
Nt: -
20min: 459 p
1h: 278 p
4h: 559 p
Middle P only
Nt: -
20min: -
1h: -
4h: 44 p
Stalled PRE+ like
Nt: 69 p
20min: 125 p
1h: 383 p
4h: 591 p
Stalled Decoding like
Nt: 565 p
20min: 668 p
1h: 840 p
4h: 1058 p
Collided Non rot2+
Nt: 261 p
20min: 264 p
1h: 365 p
4h: 209 p
Collided Rot2+
Nt: 458 p
20min: 367 p
1h: 964 p
4h: 1810 p
B
ribosomes with close neighbors
% of total 80S
60
50
40
30
20
10
0
nt
20min
1h
4h
4h
4h
MEF
Wt
ZNF598 KO
U2OS
C
Untreated
1353 particles
5%
18%
32%
40%
20min ANS
1883 particles
13%
22%
18%
6%
32%
1h ANS
2830 particles
13%
10%
14%
34%
30%
4h ANS
4271 particles
5%
13%
14%
42%
25%
Stalled PRE+-like
Stalled decoding-like
Middle helical
Collided rotated-2+
Collided unrotated
D
Front view
Collided
Stalled
ER membrane
E
Top view
F
G
EDF1
untreated
H
EDF1
GCN1-bound collided
I
Superposition top view
J
Superposition front view
K
Model: standard collision geometry
L
Model: helical geometry
M
Collided disome with GCN1
GCN1
P1
N
Model helical geometry with GCN1
P1
GCN1
~90Å
O
Superposition

Supplementary Figure S4; Analysis of collision diversity (related to Figure 3)

(A) 3D classification workflow and final classes subtomogram averages. The classes with 2 close neighbors appearing as slightly less defined densities are boxed in cyan. The classes corresponding to typical leading ribosomes in a disome are boxed in dark blue and the ones corresponding to typical collided ribosomes are boxed in dark purple. The same class is shown in 3 different views in column: a top view, the same view clipped for visualization of the internal tRNA, and a side view to visualize the GAC. (B) Rough quantification of the abundance of ribosomes with close neighbors as estimated by 3D classification in RELION. Bar and whiskers are mean and s.d. across tomograms (untreated n = 87, 20 min ANS n = 68, 1h ANS n = 36, 4h ANS n = 45). (C) Relative abundances of the different classes visualized in (A). (D-E) Subtomogram average of ER-bound collisions in front view (D) or top view (E). ER membrane is depicted in light cyan. (F) Close-up view of mapped back ER-bound collided disomes. (G) Close-up view of the region below 40S head in untreated rotated-2 subtomogram average (light gray). A model for EDF1/Mbf1 is displayed in blue ribbon. (H) Same region with superposed subtomogram average of collided ribosome bound by GCN1 (light mauve). (I-J) Superposition of the subtomogram averages of ribosomes with a collided neighbor (in white) or 2 helical neighbors in cyan and pink in top view (I) and front view (J). (K) Model of a chain of 5 ribosomes in the collision geometry, displaying clashes for n ≥ 4. (L) Model of a chain of 7 ribosomes following the helical geometry, no clash. (M) Typical collided disome with GCN1 bound. (N) Model of a disome with helical geometry and GCN1 bound to the leading ribosome (O) superposition of M and N, revealing incompatibility of the helical geometry with GCN1 simultaneous binding to the trailing ribosome.

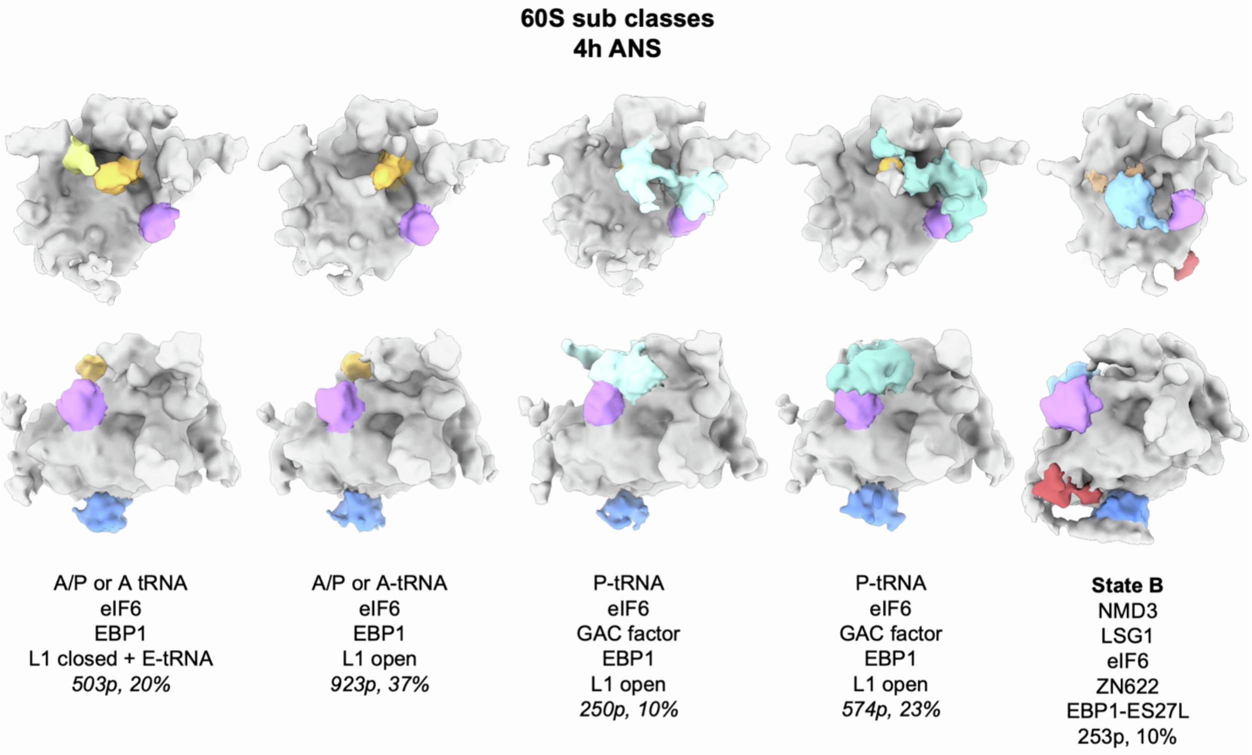

Supplementary Figure S5: 3D classes of 60S complexes in the 4h low dose ANS stress dataset (related to Figure 4)

2 views are shown in column for each class: a top view and a side view. Presence of tRNAs and extra factors bound is indicated, as well as the observed position of the L1 stalk, the amount of particles in each class and its relative abundance.

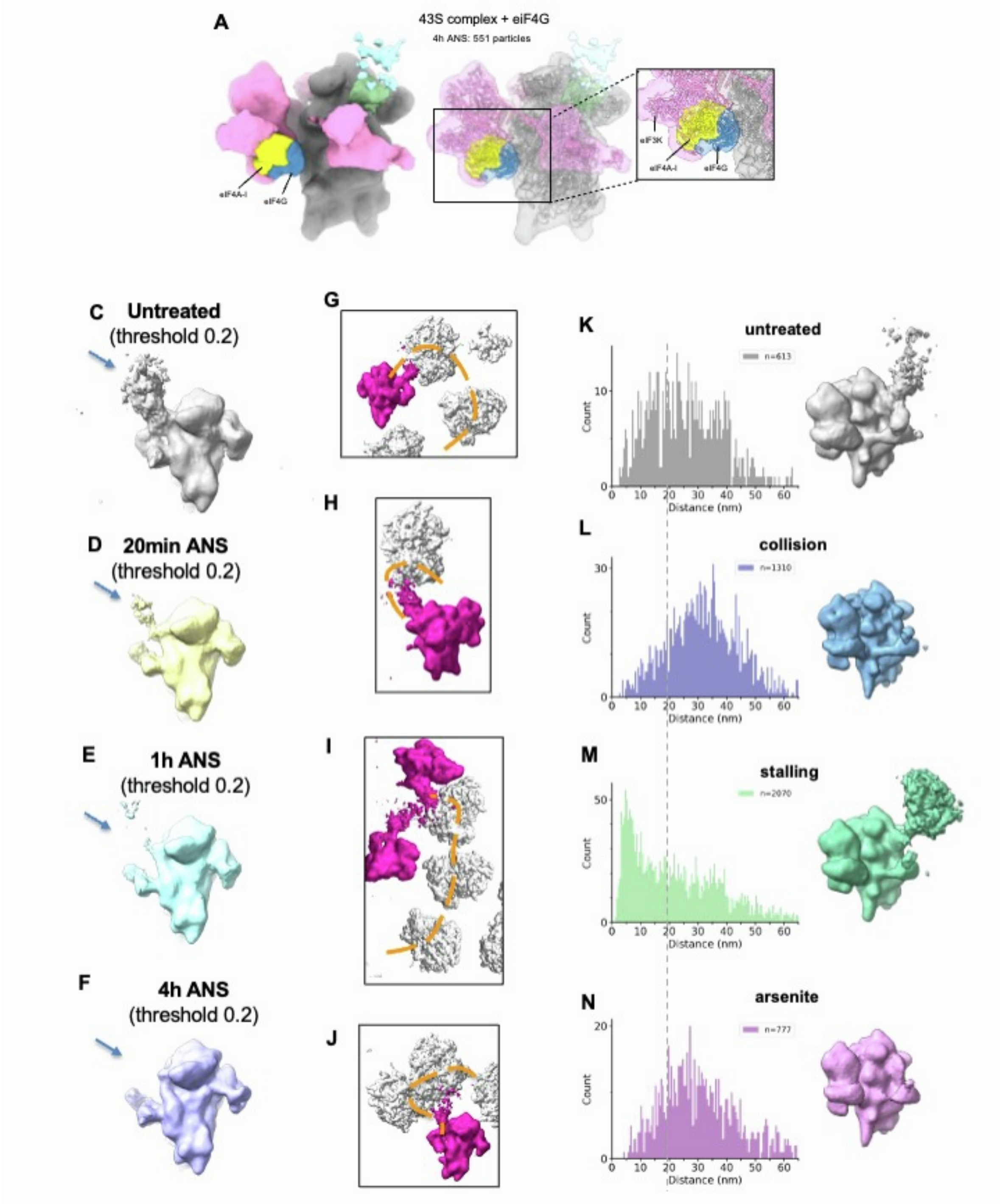

Supplementary Figure S6: Analysis of translation initiation complexes (related to Figure 5)

(A) Subtomogram average of a 4h ANS 43S subclass. The density corresponding to eIF4A1 is displayed in yellow and eIF4G in dark blue. Fitted PDB coordinates: 6ZMW.

(B) Example of 43S particles with 80S neighbors in tomograms from the control MEF cell dataset. 43S particles are displayed in pink, 80S in gray and putative mRNA is traced in dashed orange line.

(C) Analysis of mRNA density on 43S complexes. Subtomogram average of 43S complexes in (C) untreated cells, (D) 20 min ANS, (E) 1h ANS, (F) 4h ANS.

(G-J) Distance to nearest 80S neighbor of 43S particles plot and corresponding 43S subtomogram average in (G) the control untreated MEF dataset, (H) low dose ANS collision stress, (I) high dose ANS stalling stress, (J) arsenite stress. On each plot n indicates the number of distances counted in the plot.
